# Formation and Performance of a Polymer–Cement Composite Gel in Magnesium Phosphate Cement Grouting Materials Modified by Steel Slag and Latex Powder

**DOI:** 10.3390/gels12060455

**Published:** 2026-05-22

**Authors:** Jingwei Zhang, Aolin Zhang, Jia Li

**Affiliations:** 1 School of Civil Engineering, Zhengzhou University, Zhengzhou 450001, China; zhangjingwei@zzu.edu.cn (J.Z.); zal194393@163.com (A.Z.); 2 School of Water Conservancy and Transportation, Zhengzhou University, Zhengzhou 450001, China

**Keywords:** magnesium phosphate cement, grouting materials, steel slag, compressive strength, corrosion resistance

## Abstract

Magnesium phosphate cement (MPC) shows great potential for complex underground environments due to its rapid-hardening and early-strength properties. However, its large-scale application is hindered by several drawbacks, including high hydration heat, rapid setting, and insufficient long-term durability. To address these limitations, this study developed a novel MPC grouting material modified with steel slag (SS) and redispersible latex powder (LP). We systematically investigated the workability, mechanical properties, durability, and microstructural evolution of this modified system. Results indicate that incorporating SS and LP decreases both the fluidity and setting time of the grout. An optimal SS dosage accelerates reaction kinetics and raises the peak hydration temperature. Conversely, the LP-induced polymer film suppresses the overall temperature rise, delaying the first exothermic peak and advancing the second. The incorporation of 5% steel slag increased the 28-day compressive strength of the MPC to 54.86 MPa. Building on this, the combined addition of 0.15% latex powder further elevated the strength to 58.82 MPa. Microstructural and pore analyses confirmed that the steel slag enhanced interfacial bonding through physical filling and the formation of calcium phosphate crystals. Meanwhile, the latex powder formed a continuous polymer film, which tightly wrapped and bridged the hydration products and unreacted particles. This synergistic mechanism effectively sealed the capillary pores and reduced the proportion of harmful pores by 15.99% compared to the control group. Consequently, the densified MPC matrix laid a solid microstructural foundation for the material’s excellent durability. It offers reliable, high-performance material for seepage control and strata reinforcement in complex environments.

## 1. Introduction

In underground engineering, grouting is a critical technique for seepage control, settlement mitigation, and ensuring long-term structural integrity [1,2]. The effectiveness of this process is fundamentally governed by the rheological and setting behaviors of the grout material. An ideal grout must undergo rapid gelation and setting to immediately seal water ingress, while maintaining structural stability and chemical resistance in aggressive environments (e.g., chloride-rich groundwater) [3]. Magnesium phosphate cement (MPC), an acid-base binder synthesized from dead-burned magnesium oxide (MgO) and soluble phosphates, has emerged as a promising candidate. A typical MPC formulation consists of MgO, phosphate, a retarder, and various modifiers. Potassium dihydrogen phosphate (KH_2_PO_4_, KDP) is the most frequently utilized phosphate, with the primary hydration reaction given by:

(1) $$MgO+KH_{2}PO_{4}+5H_{2}O\to MgKPO_{4}\cdot 6H_{2}O$$

While MPC is widely classified as a chemically bonded ceramic, its initial hydration heavily relies on a transient sol–gel transition. Before the massive crystallization of K-struvite (MgKPO_4_∙6H_2_O), an amorphous gel-like precursor forms, dictating the early-age fluidity and structural build-up [4,5,6]. Despite its rapid hardening and high early strength, the practical application of MPC faces bottlenecks: prolonged water exposure triggers the hydrolysis of K-struvite, while the high water-to-binder ratios required for injectability induce elevated porosity and drying shrinkage [7,8,9,10]. Moreover, in complex underground projects, grouting materials are frequently subjected to the dual disturbances of groundwater hydrodynamics and dynamic engineering loads. Consequently, these materials must possess not only rapid hardening and high early strength but also superior impermeability and deformation coordination [11].

To overcome these inherent defects and meet stringent engineering requirements, researchers have extensively modified MPC grouts using mineral admixtures and organic polymers. Among various solid wastes, steel slag (SS) exhibits significant potential. Beyond acting as a micro-aggregate to refine macropores, the free CaO in SS reacts chemically with phosphate ions to generate amorphous calcium-bearing phosphate gels [12,13]. These gel phases effectively fill capillary micro-cracks and densify the interfacial transition zone (ITZ), enhancing the long-term bearing capacity and water resistance of the MPC matrix [14]. Concurrently, to mitigate the inherent high brittleness and poor deformation compatibility of pure MPC, organic polymers such as redispersible latex powder (LP) have been introduced. During the hydration and subsequent hardening of the cementitious matrix, the latex powder particles coalesce to form a continuous and flexible polymer film [15]. This organic network intertwines with the inorganic hydration products, significantly improving the bond strength, deformation capacity, and water resistance of the matrix.

Despite the documented benefits of these individual modifiers, critical technological gaps remain. Previous studies have predominantly focused on single-component modifications, which inevitably entail performance trade-offs. While mineral admixtures like SS improve matrix compactness and long-term strength, they cannot overcome the inherent brittleness and poor deformability of MPC. Consequently, its application is severely limited in grouting reinforcement scenarios that require withstanding dynamic loads and complex deformations [16,17,18]. On the other hand, the sole incorporation of polymers like LP, though highly effective in imparting flexibility, often severely retards hydration kinetics and compromises early compressive strength due to the barrier effect of the polymer film [19,20,21]. More importantly, the complex physical-chemical interactions when both an inorganic solid waste (SS) and an organic polymer (LP) are co-incorporated remain largely unexplored. It is entirely unclear how the dynamic organic polymer gel film interacts with the amorphous inorganic calcium/magnesium phosphate gels during the early hydration phase, and whether they can synergistically mitigate each other’s deficiencies. This lack of a clear cross-scale collaborative mechanism severely hinders the targeted design of high-performance MPC grouts for demanding subterranean environments.

To address this research gap, this study introduces a novel “rigid-flexible” synergistic modification strategy. Breaking away from traditional single-component modification, its primary innovation is uncovering the multi-scale chemical reactions and synergistic reinforcement mechanisms of the SS and LP co-blended system. The workability, hydration kinetics, compressive strength, and long-term durability (water and chloride erosion resistance) are systematically evaluated to clarify the SS-LP coupling effects. Furthermore, macroscopic testing is combined with micro-characterizations (XRD, pore structure analysis, and SEM) to elucidate how inorganic gels and organic films intertwine into a dense interpenetrating network. Ultimately, these findings lay a crucial theoretical and practical groundwork for utilizing modified MPC composite grouts for seepage control and strata reinforcement in complex, water-rich geological environments.

## 2. Results and Discussion

### 2.1. Workability

**Figure 1:**
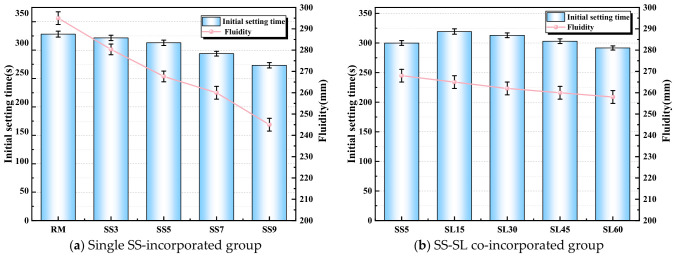
Setting time and fluidity of MPC grouting materials.

As shown in Figure 1a, increasing the SS dosage from 0% to 9% leads to a monotonic decrease in both the initial setting time and fluidity of the MPC paste. The setting time and fluidity of the reference mixture (RM) are 315 s and 295 mm, respectively. In contrast, the SS9 group shows values of 262 s and 245 mm, reflecting reductions of 16.8% and 17.1%. These macroscopic workability parameters directly reflect the early hydration kinetics of the MPC grouts. The setting time serves as an indirect indicator of the hydration rate, with faster reactions accelerating the setting process [22]. Fluidity, on the other hand, is mainly controlled by early hydration products. The massive precipitation of these products allows microscopic crystals to rapidly intertwine x cinto a spatial network, causing a macroscopic drop in fluidity. Thus, the data in Figure 1a suggest that SS addition accelerates MPC hydration. Additionally, the excess SS particles act as inert fillers, further increasing the slurry viscosity and restricting its flow [23].

In contrast, Figure 1b demonstrates that adding LP to the 5% SS baseline mixture continuously reduces the paste fluidity. The initial setting time, however, first increases and then drops sharply. Specifically, the setting time and fluidity of the LP-free group (SS5) are 300 s and 268 mm. The setting time peaks at 319 s with a 0.15% LP dosage, corresponding to fluidity of 265 mm. At a 0.6% LP dosage, these values fall to 292 s and 258 mm, marking decreases of 3.1% and 3.7% from the SS5 baseline. These trends likely stem from two distinct mechanisms. At low concentrations, LP forms a physical barrier that hinders ion migration, thus delaying hydration [21]. At higher concentrations, the extensively intertwined polymer chains within the matrix may serve as nucleation substrates. These chains offer abundant attachment sites for struvite crystals, thereby accelerating the crystallization process to a certain extent [24,25].

### 2.2. Hydration Temperature

Based on previous studies regarding the hydration of MPC [26], the MPC hydration process can be broadly divided into two stages according to the timing of the exothermic peaks. The initial stage primarily involves the dissolution of MgO and KDP. Upon dissolution in water, KDP rapidly releases K^+^, H^+^ and phosphate ions. Driven by the H^+^, MgO undergoes hydrolysis, gradually generating Mg^2+^ and OH^−^. The primary reaction equations are as follows:

(2) $$KH_{2}PO_{4}\to K^{+}+H_{2}PO_{4}^{-}$$

(3) $$H_{2}PO_{4}^{-}\to H^{+}+HPO_{4}^{2-}$$

(4) $$MgO+H^{+}\to Mg^{2+}+OH^{-}$$

The second stage primarily involves the extensive nucleation and crystal growth of K-struvite. This stage is dominated by highly exothermic precipitation, which forms a cementitious network and drives rapid strength development. The primary reaction equations are as follows:

(5) $$Mg^{2+}+K^{+}+HPO_{4}^{2-}+6H_{2}O\to MgKPO_{4}\cdot 6H_{2}O↓+H^{+}$$

**Figure 2:**
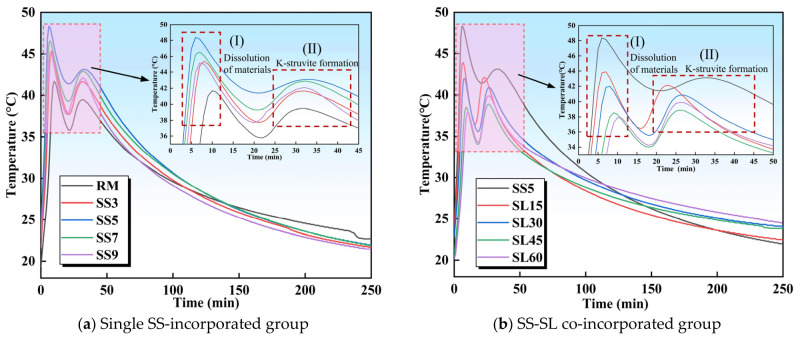
Effects of steel slag and polymer powder on the hydration temperature of MPC grouting materials.

As shown in Figure 2, the incorporation of SS and LP significantly alters the hydration heat evolution of the MPC grouts. Figure 2a,b reveal two distinct exothermic peaks (I and II) during the early hydration stage. These two peaks correspond to the two primary phases of the MPC hydration process, namely the dissolution of raw materials and the massive precipitation of struvite crystals.

As shown in Figure 2a, the temperatures of both exothermic peaks for the SS-modified MPC systems are consistently higher than those of the reference mixture (RM). As the SS dosage increases, these peak temperatures exhibit an initial increase followed by a decrease. Incorporating a moderate amount of SS introduces free calcium oxide (f-CaO). The intense exothermic hydrolysis of f-CaO, combined with its chemical activation on MPC hydration, significantly elevates the initial temperature and heat release rate. This observation aligns perfectly with the macroscopic workability results, confirming that SS accelerates hydration. However, at higher SS dosages (7% and 9%), the relative proportion of the MPC matrix decreases. Consequently, the inert filler effect of excess slag particles becomes dominant. This effect hinders ion interactions and restricts the continuous growth of the hydration product network, ultimately reducing the exothermic peak values [27,28,29].

As Figure 2b shows, adding LP to the 5% SS baseline mixture reduces the overall hydration temperature. LP delays and weakens the first exothermic peak, while advancing and weakening the second peak. The first peak is delayed primarily because LP forms a coating around the MgO particles, restricting early ion dissolution [30]. Although this coating hinders magnesium ion diffusion and causes local supersaturation—thereby triggering early struvite nucleation and advancing the second peak—the polymer film ultimately inhibits the overall reaction of active ions. This leads to a macroscopic decrease in total hydration heat and peak temperatures [31]. These thermal results, highlighting delayed dissolution and suppressed heat generation, perfectly confirm the macroscopic observation that moderate LP addition prolongs the setting time.

### 2.3. Compressive Strength

**Figure 3:**
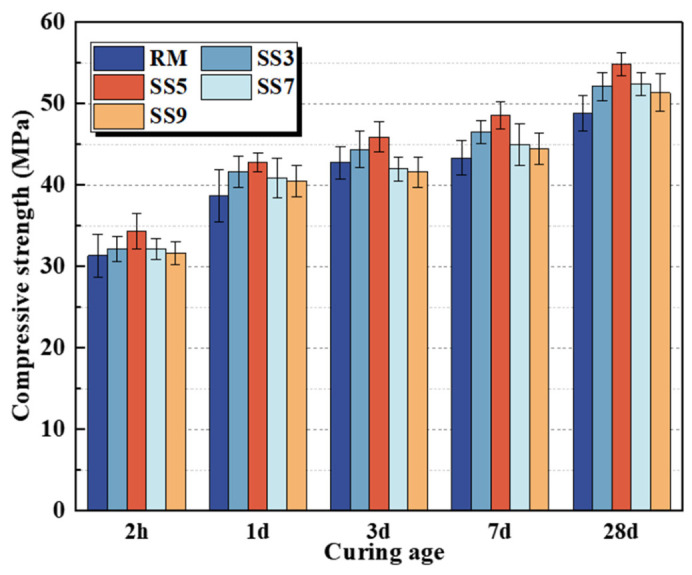
Compressive strength of MPC grouting materials with single addition of SS.

Figure 3 presents the compressive strength evolution of the MPC system with different SS mass fractions (0–9%). At all tested ages, the compressive strength increases with SS dosage up to 5%, where it reaches its peak, and then decreases at higher dosages (7% and 9%). For instance, the 28 d compressive strengths of the SS-modified groups are 52.12, 54.86, 52.39, and 51.33 MPa, which are 6.7%, 12.3%, 7.25%, and 5.08% higher than that of the reference group, respectively. This confirms that an optimal 5% SS addition significantly strengthens the MPC, while excessive SS degrades its mechanical performance.

This phenomenon stems from both physical and chemical mechanisms. Physically, the 38 μm SS particles act as micro-aggregates to optimize particle gradation and reduce porosity [28]. Chemically, the active f-CaO in SS hydrates and releases calcium ions, which then react with residual phosphate ions to form cementitious calcium phosphate. These precipitates fill the pores and densify the matrix [29,32]. However, when the SS dosage is too high, a dilution effect occurs. The reduced proportion of active-matrix materials leads to fewer hydration products, which weakens the load-bearing skeleton and causes the subsequent strength decline.

**Figure 4:**
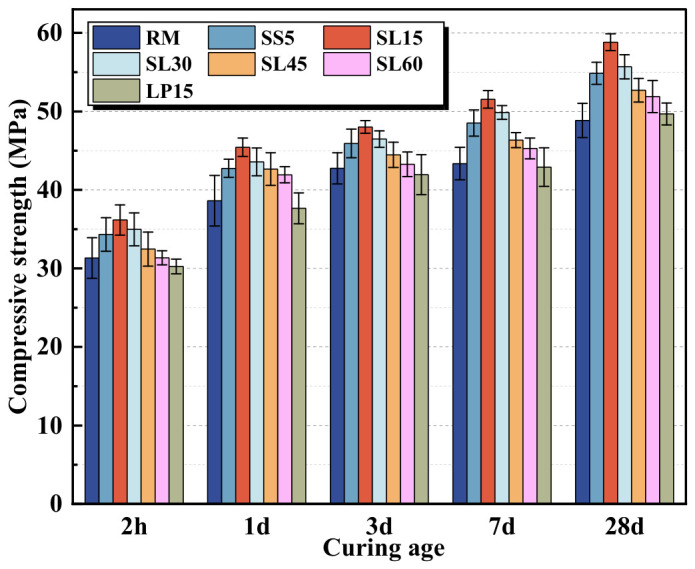
Compressive strength of MPC grouting materials with single addition of SS and LP.

As Figure 4 illustrates, the compressive strength of the 5% SS-based MPC composite modified with LP shows a trend like that of the single SS-blended system. A 0.15% LP addition yields a peak 28 d compressive strength of 58.82 MPa, a 7.22% improvement over the SS5 baseline. This confirms the synergistic strengthening effect of combining an optimal amount of LP with SS. Additionally, LP significantly impacts late-stage strength development. Between 3 d and 7 d, the compressive strengths of the SS5, SL15, and SL30 groups rise from 45.92, 48.01, and 46.48 MPa to 48.52, 51.52, and 49.87 MPa (increases of 5.7%, 7.2%, and 7.3%). At 28 d, these values further increase by 13.1%, 14.2%, and 11.7% from their 7 d levels, reaching 54.86, 58.82, and 55.68 MPa, respectively. These data clearly show that LP promotes late-stage hydration and strength evolution. Nevertheless, excessive LP introduces negative effects. It substantially increases paste viscosity and hinders ion migration. Moreover, the low-modulus polymer networks encroach upon the space required by the rigid load-bearing phase, which ultimately degrades the macroscopic compressive strength [33].

Furthermore, comparing the compressive strengths of the RM, SS5, SL15, and LP15 groups at identical curing ages reveals a distinct synergistic effect. While the single addition of 5% SS enhances strength, adding LP alone negatively impacts early strength. However, their composite addition yields a higher overall strength than the SS5 group. This synergy stems from complementary mechanisms: the accelerating and micro-aggregate effects of the SS perfectly offset the retarding and air-entraining drawbacks of the LP. Conversely, the flexible polymer network formed by the LP effectively mitigates the inherent brittleness of the SS-MPC matrix. Together, these complementary advantages significantly optimize the overall mechanical performance of the material.

### 2.4. Corrosion Resistance

#### 2.4.1. Water Resistance

**Figure 5:**
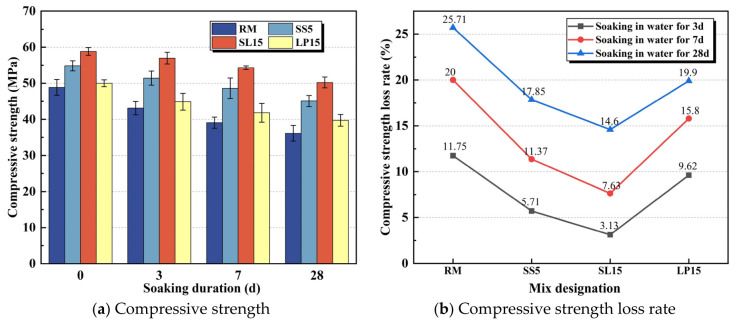
Strength variations of specimens at different ages after water immersion.

Figure 5 illustrates the compressive strength evolution of the MPC grouts subjected to varying durations of water immersion. As shown in Figure 5a, prolonged water exposure inevitably causes mechanical degradation across all groups. However, the strength loss rate curves (Figure 5b) indicate that incorporating SS and LP significantly mitigates this deterioration. Specifically, after 28 days of immersion, the strength loss rates for the specimens with solely 5% SS and 0.15% LP are restricted to 17.85% and 19.90%, respectively. Notably, the composite addition of 0.15% LP to the 5% SS baseline further reduces the 28-d loss rate to 14.60%, achieving optimal water resistance. This demonstrates a pronounced synergistic effect between SS and LP in resisting water degradation and maintaining structural integrity.

#### 2.4.2. Salt Corrosion Resistance

**Figure 6:**
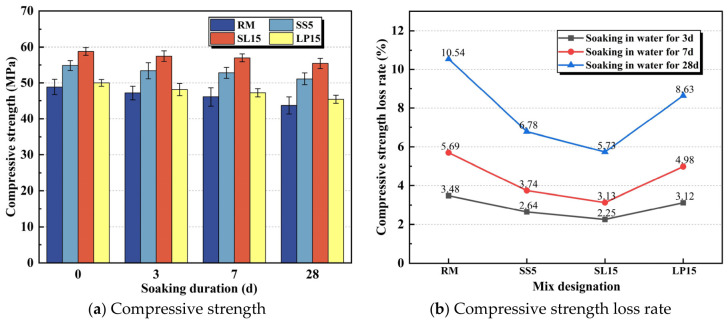
Compressive strength variations under NaCl solution attack.

As shown in Figure 6, after 28 days of continuous exposure to a 5% NaCl solution, all MPC specimens experience varying degrees of mechanical degradation but maintain robust resistance to salt attack. The compressive strengths of the RM, SS5, SL15, and LP15 groups remain at 41.36 MPa, 51.14 MPa, 55.45 MPa, and 45.38 MPa, respectively. Notably, the SL15 group exhibits exceptional resistance, recording a 28-d strength loss rate of only 5.73%—a 45.6% reduction compared to the RM group (10.54%). This macroscopic improvement stems fundamentally from a synergistic densification: the physical filling by SS micro-aggregates and the effective sealing of capillary pores by the polymer film. Together, they sever the interconnected pore network within the matrix, effectively hindering the penetration and migration of free chloride ions. Consequently, this composite material is highly suitable for long-term service in complex saline groundwater environments.

### 2.5. Pore Structure Analysis

**Figure 7:**
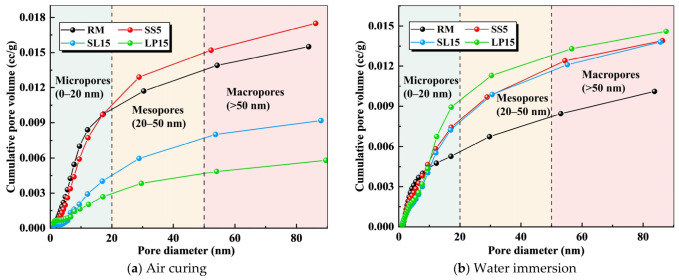
Pore size distribution of specimens under different curing conditions.

As shown in Figure 7, nitrogen adsorption reveals that SS-modified specimens exhibit similar or slightly higher total pore volumes compared to the reference group under air-curing and water-immersion. This apparent contradiction with their enhanced macroscopic strength occurs because micron-sized SS particles primarily fill micrometer-scale, rather than nanometer-scale, pores. These hard particles establish a rigid micro-skeleton that boosts overall load-bearing capacity, effectively offsetting any negative impact from nano-pore fluctuations. Conversely, incorporating LP drastically reduces the total pore volume from 0.016 cc/g to 0.006 cc/g.

**Figure 8:**
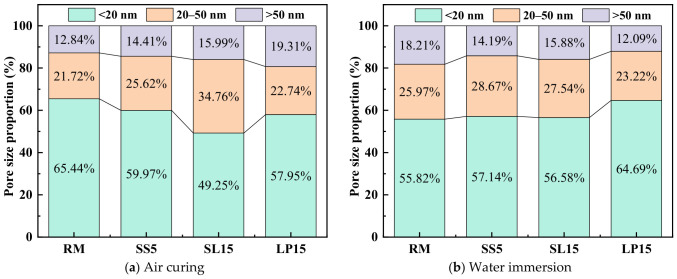
Pore size fractions of specimens under different curing conditions.

As shown in Figure 8, the SL15 group exhibits superior mechanical properties to LP15 despite a slightly higher total pore volume, indicating total porosity is not the sole limiting factor in dense systems. Based on pore classification [34,35], SL15 contains significantly more less-harmful mesopores (20–50 nm) at 34.76% and fewer harmful pores (>50 nm) at 15.99%, compared to the 19.31% in LP15. This microstructural enhancement occurs because incorporated SS refines large macropores into fine mesopores, significantly increasing the tortuosity of the pore network.

Following water immersion, the total pore volume of the reference group (RM) decreases, yet the fraction of harmful pores increases by 5.37%. Although moisture ingress triggers the rehydration of unreacted MgO and dissolved phosphates, the resulting crystalline products are insufficient to offset the surge in macropores caused by localized leaching. For the LP15 group, the harmful pore fraction remains relatively constant after prolonged immersion, but the total pore volume increases to 0.014 cc/g, primarily due to a rise in gel pores. Notably, the proportion of harmful pores drops from 19.31% to 12.09%, which is attributed to the swelling of the polymer film. The hydrophilic latex film absorbs water and swells during long-term immersion; this volumetric expansion creates intermolecular gaps within the originally dense film, thereby redistributing the pore structure.

### 2.6. Hydration Product and Microstructural Analysis

#### 2.6.1. XRD Analysis

**Figure 9:**
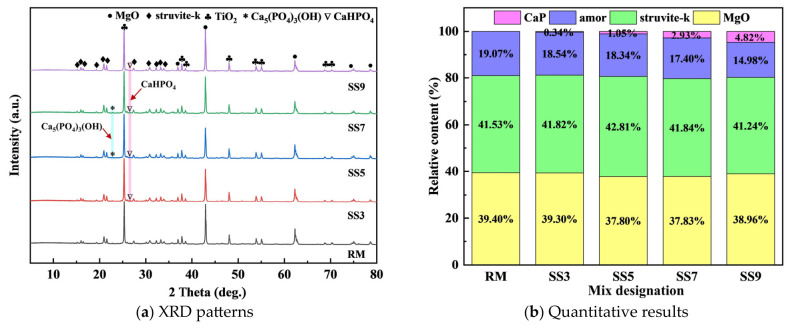
Analysis of hydration products for MPC with single addition of steel slag after 28 d of air curing.

Figure 9 presents the XRD patterns of the SS-modified groups at 28 days. Distinct diffraction peaks for calcium phosphates are observed, indicating a reaction between the steel slag and phosphate ions. Rietveld refinement results (Figure 9b) reveal that the residual MgO content initially decreases and then increases with higher SS dosages. At a 5% dosage, the hydration product content peaks at 42.81%, consistent with the compressive strength trend and confirming the decisive role of hydration products in mechanical performance. At low dosages, the alkaline components in SS hydrolyze and elevate the pH, facilitating MgO dissolution and K-struvite formation. Conversely, beyond 5% SS, a competitive effect dominates. Abundant dissolved Ca^2+^ competes with Mg^2+^ for phosphate ions, leading to reduced K-struvite content and increased residual MgO. Because K-struvite directly governs the compressive strength of MPC, this explains the strength reduction observed at high SS dosages. The chemical reactions involved are presented below:

(6) $$Ca^{2+}+HPO_{4}^{-}+2H_{2}O\to CaHPO_{4}\cdot 2H_{2}O↓$$

**Figure 10:**
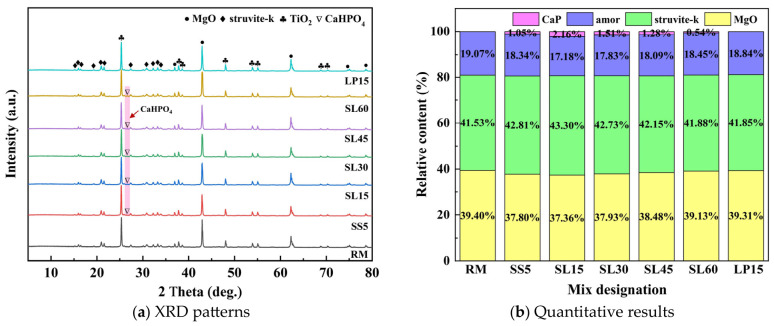
Hydration product analysis of the SS-LP composite system after 28 d of air curing.

Figure 10 illustrates the XRD patterns of the SS-LP composite system after 28 d of curing. At a constant 5% SS content, the intensity of MgO diffraction peaks increases with higher LP dosages. Notably, the incorporation of 0.15% LP (LS15) significantly enhances the intensity and sharpness of the primary hydration product, K-struvite. This suggests that an appropriate LP addition improves the local crystallization environment, facilitating struvite lattice development and crystal growth. As shown in Figure 10b, the LS15 group achieves the highest K-struvite content (43.30%), further confirming the decisive role of hydration product volume in mechanical performance.

A comparison of the 28-day XRD patterns for the RM and LP15 groups reveals significantly more prominent K-struvite diffraction peaks in the latter. This intensification suggests that the incorporation of latex powder (LP) effectively enhances late-age strength development. This film not only constructs an interconnected network structure but also retains a small amount of moisture, thereby providing an internal curing effect within the system.

#### 2.6.2. SEM Analysis

**Figure 11:**
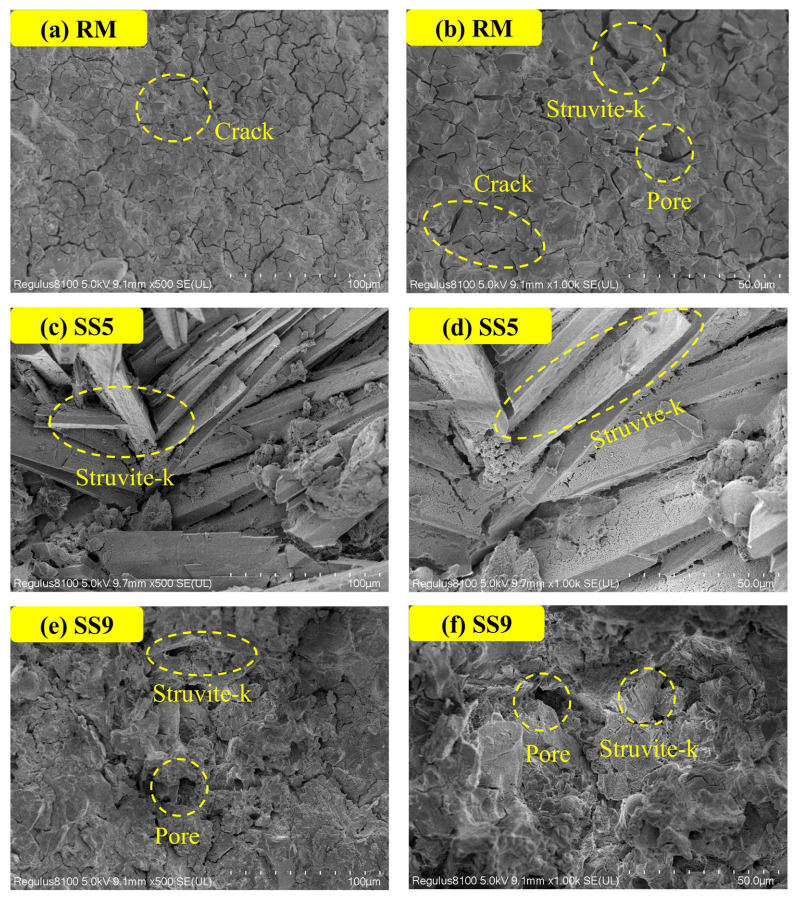
Micro-morphology of MPC grout modified with SS alone after 28 d of air curing.

Figure 11 illustrates the microstructure of MPC with various steel slag dosages. The control group (Figure 11a,b) exhibited a porous and discontinuous matrix, primarily composed of disordered, irregularly shaped short columnar or hexagonal K-struvite crystals. The prevalence of interstitial micropores and microcracks significantly compromised internal cohesion, fundamentally accounting for the inferior macroscopic mechanical strength of this group. In contrast, the 5% SS group (Figure 11c,d) showed substantial microstructural optimization. Coarser, well-crystallized tabular and elongated columnar K-struvite crystals interlocked to form a dense and robust three-dimensional load-bearing framework. Furthermore, irregularly shaped crystals were observed filling the cross-sectional microstructure. These are newly formed calcium-bearing phosphates, generated by the reaction between free CaO in the SS and phosphate ions. They effectively enhance interfacial filling and cementation, directly corroborating the calcium phosphate species identified in the XRD patterns.

However, increasing the SS content to 9% (Figure 11e,f) led to evident microstructural degradation. The K-struvite crystals transitioned from robust columns/plates to fine, flattened flakes, accompanied by a rebound in porosity. This is primarily attributed to the intense competitive effect of excess Ca^2+^, which disrupts the crystallization pathway of K-struvite. The resulting decrease in crystallinity and feeble inter-crystalline interlocking failed to establish an effective stress-transfer skeleton, revealing the micro-mechanism underlying the strength regression at high SS dosages.

**Figure 12:**
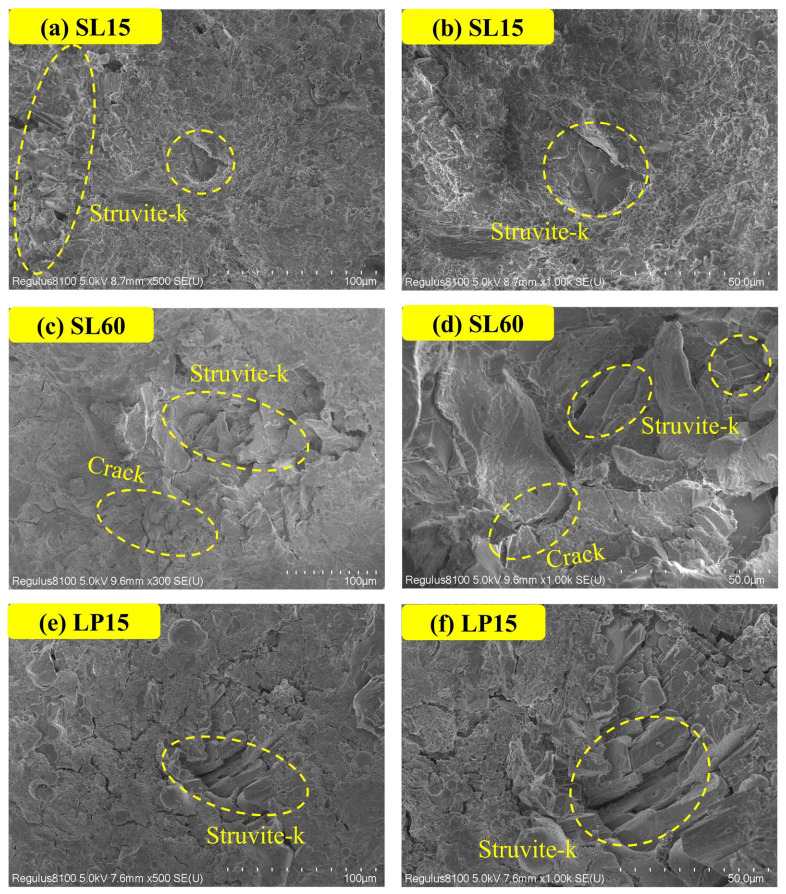
Micro-morphology of the SS-LP composite modified MPC grout after 28 d of air curing.

Figure 12 illustrates the microstructure of MPC with the combined addition of SS and LP. Incorporating an optimal amount of LP into the 5% SS-based system (Figure 12a,b) achieves ultimate densification, resulting in an exceptionally smooth fracture surface devoid of discernible microcracks. This superior morphology is primarily attributed to the continuous flexible polymer film formed via LP demulsification. This film fills capillary pores and provides vital crack-bridging and interfacial bonding, transforming the brittle inorganic skeleton into a robust inorganic-organic interpenetrating network. Such a structure significantly enhances matrix toughness and suppresses crack initiation at the source.

Conversely, exceeding the optimal LP threshold (Figure 12c,d) triggers a sharp increase in slurry viscosity, which hinders the long-range migration and diffusion of aqueous ions. This restricted ion transport induces localized heterogeneous hydration and impairs K-struvite development, ultimately leading to fragmented hydration products, loose packing, and the recurrence of microcracks. Furthermore, in the system modified with LP alone (Figure 12e,f), the fracture surface remains densely populated with shrinkage cracks, indicating that individual polymer addition is insufficient to suppress early-age volumetric shrinkage. Although LP provides heterogeneous nucleation sites that facilitate localized crystal growth, the absence of the SS micro-filler effect leaves macroscopic defects unaddressed. These micro-scale observations confirm a synergistic effect between SS and LP, which significantly densifies the MPC matrix at appropriate dosages.

### 2.7. Synergistic Mechanism of SS and LP Modification

**Figure 13:**
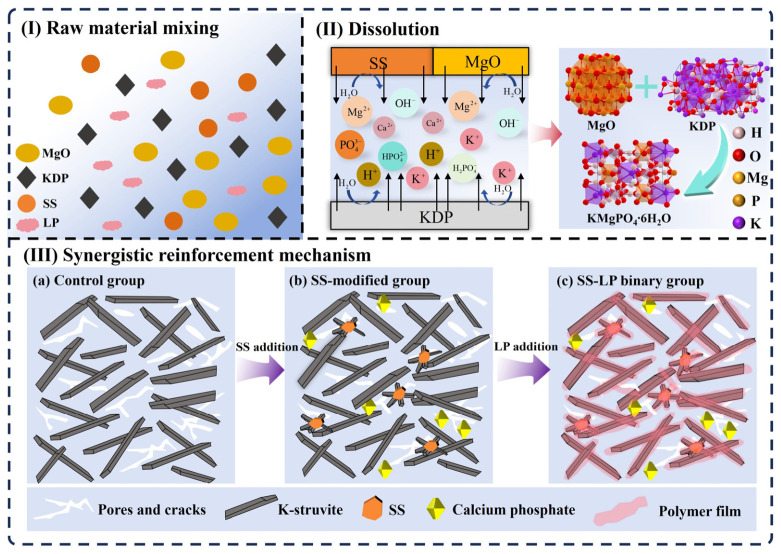
Synergistic modification mechanism of steel slag and latex powder.

The synergistic modification mechanism of the SS-LP composite system manifests as a rigid-flexible composite mechanism. As illustrated in Figure 13, it integrates the rigid reinforcement of the SS skeleton with the flexible network filling effect provided by LP. The underlying mechanism can be summarized as an evolutionary process across three dimensions: physical space, chemical interface, and mechanical response.

#### 2.7.1. Physical Space Reconstruction

The unmodified MPC matrix contains numerous interconnected pores due to the loose packing of its hydration products. In the composite system, however, the micro-scale SS particles act as micro-aggregates. They preferentially fill the large capillary pores and establish a rigid load-bearing skeleton within the matrix. Concurrently, the LP particles demulsify and coalesce during the hydration process to form a continuous polymer film. This flexible film further seals the micro-defects between inorganic particles and interweaves through the crystal network. Consequently, a dense interpenetrating structure of the inorganic skeleton and organic film is constructed in the physical space. This densified microstructure endows the MPC composites with excellent resistance to water and chloride ion erosion.

#### 2.7.2. Multiphase Chemical Interface Coupling

Compared to the reference group, the interfacial bonding state of the composite system undergoes a fundamental alteration. Active components, such as free calcium oxide (f-CaO), dissolved from the surface of the SS particles, undergo a secondary reaction with phosphate ions. The resulting amorphous calcium-bearing gels interweave with the primary K-struvite crystals. Furthermore, owing to its high adhesiveness, the LP polymer film tightly envelops both the SS particles and the inorganic crystals. The physical entanglement and adsorption between this organic network and the inorganic gels effectively fortify the inherently weak interfacial transition zone (ITZ). Consequently, the connections among the various phases are transformed from loose contacts to robust anchoring.

#### 2.7.3. Macroscopic Mechanical Response

The microstructural evolution directly dictates the load-bearing behavior of the material. Under external loading, the high-strength rigid SS skeleton acts as the primary load-bearing unit to transfer stress, thereby guaranteeing the compressive capacity of the composite. When localized stress concentration induces microcracks, the flexible polymer films spanning these cracks restrict crack-tip propagation through a bridging effect. Furthermore, the elastic tensile deformation of the polymer network effectively absorbs and dissipates fracture energy. This synergy—combining the deformation resistance of the rigid skeleton with the energy dissipation of the flexible network—mitigates the inherent brittle fracture characteristics of the matrix. Ultimately, it fulfills the strict engineering requirements for deformation compatibility in anti-seepage reinforcement within complex underground environments.

## 3. Conclusions

This study systematically investigated the effects of SS and LP dosages on the workability, hydration heat evolution, mechanical properties, and long-term durability of two-component MPC-based grouts. The underlying mechanisms governing the performance enhancement provided by SS and LP were elucidated. Based on the experimental results, the primary conclusions are as follows:

(1) The combined incorporation of SS and LP reduces the fluidity and shortens the setting time of MPC grouts. SS promotes hydration via micro-filling and nucleation effects. At the optimal dosage of 5%, SS elevates the early exothermic peak and optimizes the K-struvite crystal morphology. Conversely, LP retards heat evolution through polymer film formation and interfacial bridging, which further densifies the matrix.
(2) Co-incorporating SS and LP demonstrates a remarkable synergistic optimization effect. Under air curing conditions, the optimal combination (5% SS and 0.15% LP) yields abundant hydration products and a highly dense microstructure. Consequently, the 28-d compressive strength of this group (SL15) increases by 7.22% compared to the single SS-blended baseline (SS5). However, excessive dosages dramatically increase the system’s viscosity and hinder ion migration, leading to incomplete hydration and degraded mechanical properties.
(3) The SS-LP composite significantly refines the pore size distribution of the MPC matrix. Under air curing, the proportion of harmful pores decreases to 15.99%, while low-harm pores account for 34.76%. Following long-term water immersion, this dense micro-morphology remains intact, and the harmful pore fraction stays largely stable. This microstructural integrity provides a robust foundation for the material’s long-term durability.
(4) The modified MPC system exhibits outstanding water resistance and salt-erosion durability. The 28-d compressive strength loss after long-term water immersion is merely 14.6%, substantially overcoming the inherent water sensitivity of traditional MPC. Furthermore, under sodium chloride attack, chloride ion penetration is effectively suppressed, limiting the strength loss to just 5.73%. Consequently, this advanced composite fully satisfies the rigorous requirements for grouting reinforcement in complex underground environments.

Although this study systematically elucidates the pore structure evolution and synergistic reinforcement mechanisms of the MPC system co-modified by SS and LP, certain limitations remain, which provide clear directions for future research. First, the current durability tests were limited to a 28-day exposure in pure water and single-salt environments. This short-term approach does not fully capture the long-term degradation under the complex hydro-mechanical-chemical coupled conditions typical of underground engineering. Therefore, future studies will prioritize evaluating the long-term durability and in-situ service performance of the material under these multi-field coupled environments. Second, this study utilized macroscopic temperature evolution as an indirect indicator to qualitatively assess hydration intensity. While valuable for understanding practical setting behaviors, this approach lacks precise quantitative thermodynamic data. Consequently, future work will employ systematic isothermal calorimetry tests to quantitatively determine the total heat release, hydration activation energy, and detailed thermodynamic kinetics of the composite system. Finally, we proposed a rigid-flexible coupling theory to explain the anomalous phenomenon wherein a higher pore volume yields higher strength. However, this explanation remains largely phenomenological. Future research will utilize advanced techniques—such as in-situ X-ray micro-CT, nanoindentation, quantitative XRD, and molecular dynamics simulations—to enable an in-depth quantitative characterization of the interfacial interactions between the inorganic hydration products and organic polymer films.

## 4. Materials and Methods

### 4.1. Raw Materials

**Figure 14:**
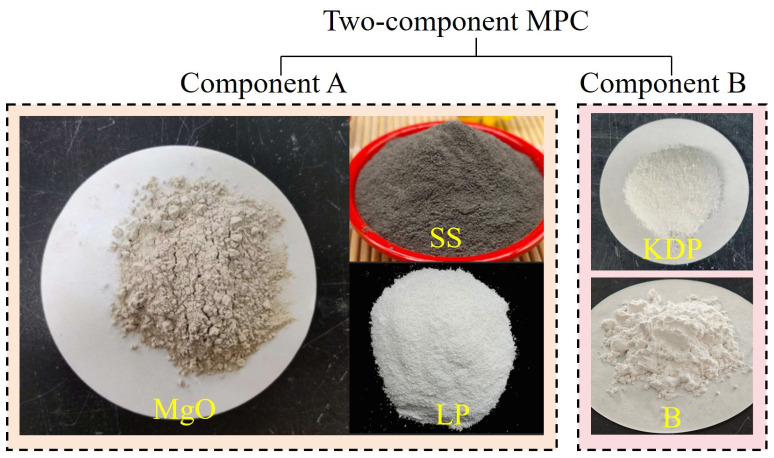
Experimental materials are used in two components.

The experimental materials used in this study include dead-burned magnesium oxide (MgO), potassium dihydrogen phosphate (KH_2_PO_4_, KDP), borax (Na_2_B_4_O_7_∙10H_2_O, B), steel slag (SS), and re-dispersible polymer powder (LP). The MPC grouting material was prepared as a two-component system, as shown in Figure 14. Component A consisted of a mixture of magnesium oxide, steel slag, re-dispersible polymer powder, and water, while Component B was a mixture of potassium dihydrogen phosphate, borax, and water.

The dead-burned magnesium oxide (MgO), featuring a density of 3.58 g/cm^3^ and an average particle size of 200 mesh, was sourced from Jubo High-Temperature Refractory Materials Co., Ltd. in Liaoning, China. The potassium dihydrogen phosphate (KDP), appearing as colorless crystals or a fine powder, was supplied by Composure Way Technology Co., Ltd. located in Beijing, China. Borax decahydrate, with a purity exceeding 99.5% and a particle density of 1.72 g/cm^3^, was utilized as a set retarder and obtained from Jubo High-Temperature Refractory Materials Co., Ltd. in Liaoning, China.

Regarding the selection of steel slag (SS) particle size, studies [23,36] utilized SS with an average diameter ranging from 30 to 40 μm. Their experimental results demonstrated that SS particles within this range can significantly optimize the pore structure of the MPC matrix and enhance its mechanical strength. Additionally, this specific fineness effectively improves the fluidity of the slurry. Based on these findings, this study selected SS particles supplied by Xuhan New Material Co., Ltd. (Shijiazhuang, China), with an average particle size of 38 μm (350–400 mesh).

The latex powder (LP), a re-dispersible copolymer powder of vinyl acetate and ethylene (particle size > 400 μm), was purchased from Wacker Chemie AG in Munich, Germany. The polymer modifier used in this study was a redispersible vinyl acetate-ethylene (VAE) copolymer powder. To ensure excellent water dispersibility, the VAE particles are encapsulated by a polyvinyl alcohol (PVA) protective colloid. According to the manufacturer’s specifications, the degree of hydrolysis of the PVA is approximately 88%. The VAE powder has a solid content of 98%, an ash content of 13%, a bulk density of 490–590 kg/m^3^, and a minimum film-forming temperature of 4 °C. This LP can be rapidly re-dispersed upon contact with water, facilitating the subsequent formation of the polymer gel network. XRD analyses of MgO and SS are presented in Table 1.

### 4.2. Sample Preparation

Grounded in extensive preliminary investigations and established findings in the literature [37,38], it was observed that the MPC achieves an optimal balance between hydration kinetics, workability, and mechanical strength when the magnesia-to-phosphate ratio (M/P) is approximately 2.0, the water-to-binder ratio (W/C) is approximately 0.22, and the borax-to-magnesia ratio (B/M) is approximately 8%. Therefore, to accurately evaluate the actual effects of the subsequently added modifiers, this classic optimized formulation was selected as the benchmark control group for all composite grouting systems.

Previous research [15] demonstrated that incorporating 0.5% LP into MPC yields superior flexural and compressive strengths compared to LP-free samples. This specific dosage also provides the highest water stability and the lowest water absorption. Based on these findings and our extensive preliminary experiments, we selected the 0.5% LP addition as a central reference point. Consequently, we designed four gradient LP dosages—0.15%, 0.30%, 0.45%, and 0.60%—to systematically evaluate the performance of the SS-LP co-modified MPC grouts.

The mix proportions for the MPC grouting materials are provided in Table 2, comprising a total of 10 groups. In these formulations, M/P specifically refers to the mass ratio of magnesium oxide to potassium dihydrogen phosphate; B/M refers to the mass ratio of borax to magnesium oxide; and W/C represents the ratio of the total mass of water to the sum of the masses of magnesium oxide and potassium dihydrogen phosphate. The mass fractions of SS and re-dispersible LP were calculated based on their respective ratios to the total mass of the cementitious materials.

### 4.3. Experimental Methods

#### 4.3.1. Properties of Fresh MPC Grouts

The setting time of the MPC was determined using a Vicat apparatus (DMYYM-1, Jinan Rutong Testing Technology Co., Ltd., Jinan, Shandong, China) in accordance with the Chinese standard GB/T 1346-2024 [39]. Given the rapid hydration kinetics of MPC and the narrow interval between initial and final setting, only the initial setting time was recorded as the representative setting time for the paste. The fluidity of the grout was measured following GB/T 8077-2023 [40]. To eliminate potential errors caused by the rapid loss of workability during repeated measurements, fresh mixtures were prepared under identical proportions and laboratory conditions for each trial. The final fluidity values were reported as the average of three independent measurements. The hydration temperature within the initial 250 min was monitored using a temperature data logger (Elitch RC-4, Xuzhou, Jiangsu, China). The measurements were conducted in a controlled environment at a temperature of 20 ± 2 °C.

#### 4.3.2. Mechanical Properties Test

**Figure 15:**
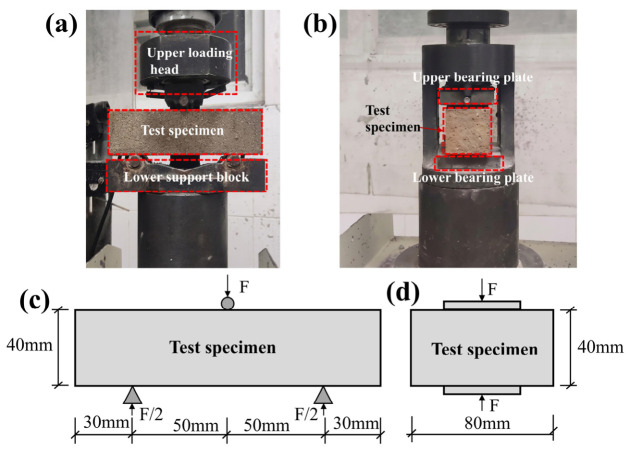
Mechanical property tests of MPC: (**a**) flexural strength test; (**b**) compressive strength test; (**c**) specimen dimensions for the flexural strength test; (**d**) specimen dimensions for the compressive strength test.

The mechanical properties of the MPC grouting materials were evaluated in accordance with GB/T 17671-2021 [41]. For the flexural strength test, prismatic specimens (40 mm × 40 mm × 160 mm) were subjected to a continuous and uniform load at a rate of 50 N/s until fracture. Subsequently, the resulting half-prisms were tested for compressive strength by applying a uniform loading rate of 2.4 kN/s until failure. The peak loads were recorded to calculate the final strengths. Figure 15 illustrates the experimental setup and specimen dimensions for the mechanical testing of MPC. The specimens were cast using standard triple-gang molds, producing three 40 mm × 40 mm × 160 mm prisms per group. For the flexural test, the span-to-depth ratio was set to 2.5 (with a span of 100 mm and a specimen depth of 40 mm). Compressive strength tests were subsequently performed on the two broken halves of each prism obtained from the flexural test. Consequently, six valid compressive strength data points were collected for each group under identical mix proportions, curing conditions, and testing ages.

#### 4.3.3. Microstructural Characterization

Prior to microstructural characterization, the MPC specimens were crushed and immersed in anhydrous ethanol for 7 days to halt the hydration process. X-ray diffraction (XRD) and scanning electron microscopy (SEM) were employed to analyze the phase composition and the microstructural morphology of the hydration products, respectively.

The XRD analysis was conducted using a D8 Advance X-ray diffractometer (Bruker, Berlin, Germany) with a scanning range of 5° to 80° at a scanning rate of 5°/min. For observation using an SEM (Regulus 8100, Hitachi High-Tech Corporation, Tokyo, Japan), the ethanol-soaked sample fragments were first dried in an oven at 40 °C. To obtain high-resolution images, the specimens were subsequently sputter-coated with a 20 nm gold layer to enhance their electrical conductivity.

## Figures and Tables

**Table 1:** Chemical compositions of raw materials (wt.%).

Raw Material	MgO	SiO_2_	CaO	Fe_2_O_3_	Al_2_O_3_	Others
MgO	92.66	2.37	1.44	1.38	0.79	1.36
SS	6.98	12.24	41.29	18.45	6.03	15.01

**Table 2:** Experimental design of SS-LP synergistically modified MPC grouting materials.

Sample	Mass Ratio				
	M/P	W/C	B/M (%)	SS (%)	LP (%)
RM	2	0.22	8	0	0
SS3	2	0.22	8	3	0
SS5	2	0.22	8	5	0
SS7	2	0.22	8	7	0
SS9	2	0.22	8	9	0
SL15	2	0.22	8	5	0.15
SL30	2	0.22	8	5	0.30
SL45	2	0.22	8	5	0.45
SL60	2	0.22	8	5	0.60
LP15	2	0.22	8	0	0.15

## Data Availability

The data that support the findings of this study are available from the corresponding author upon reasonable request.
